# Supplementary material for: Physiological activation of Aryl hydrocarbon receptor by food-derived ligands is essential for the efficacy of anti-PD1 therapy
Source: Nat Commun. 2025 Dec 2;16:10598. doi: 10.1038/s41467-025-66854-x (PMC12672584; doi:10.1038/s41467-025-66854-x)
Supplement: Supplementary file 1 — Supplementary Information [file 41467_2025_66854_MOESM1_ESM.pdf]

Supplementary Information for

Physiological activation of Aryl Hydrocarbon Receptor by food-derived ligands is essential for the efficacy of anti-PD1 therapy

Alba De Juan[#], Alice Coillard[#]....Elodie Segura[*]

# These authors contributed equally: Alba De Juan, Alice Coillard.

***Correspondence:**
Elodie Segura
elodie.segura@inserm.fr
Orcid ID: orcid.org/0000-0003-1795-1921

This file includes:

Supplementary Figure 1. Dietary AhR ligands are essential for the efficacy of anti-PD1 immunotherapy in preclinical models.

Supplementary Figure 2. Relative abundance of circulating AhR ligands after microbiota manipulation.

Supplementary Figure 3. Lack of dietary AhR ligands does not significantly modify the baseline tumor myeloid profile.

Supplementary Figure 4. Lack of dietary AhR ligands does not significantly affect the baseline tumor T cells profile.

Supplementary Figure 5. Dietary AhR ligands do not impact tumor antigen-specific immune responses.

Supplementary Figure 6. Lack of dietary AhR ligands impairs NK cells infiltration without affecting granzyme B and IFN-γ production.

Supplementary Figure 7. AhR deficiency in NK cells does not impact their functional properties.

Supplementary Figure 8. AhR regulates part of the functional response of effector CD8 T cells to anti-PD1 treatment.

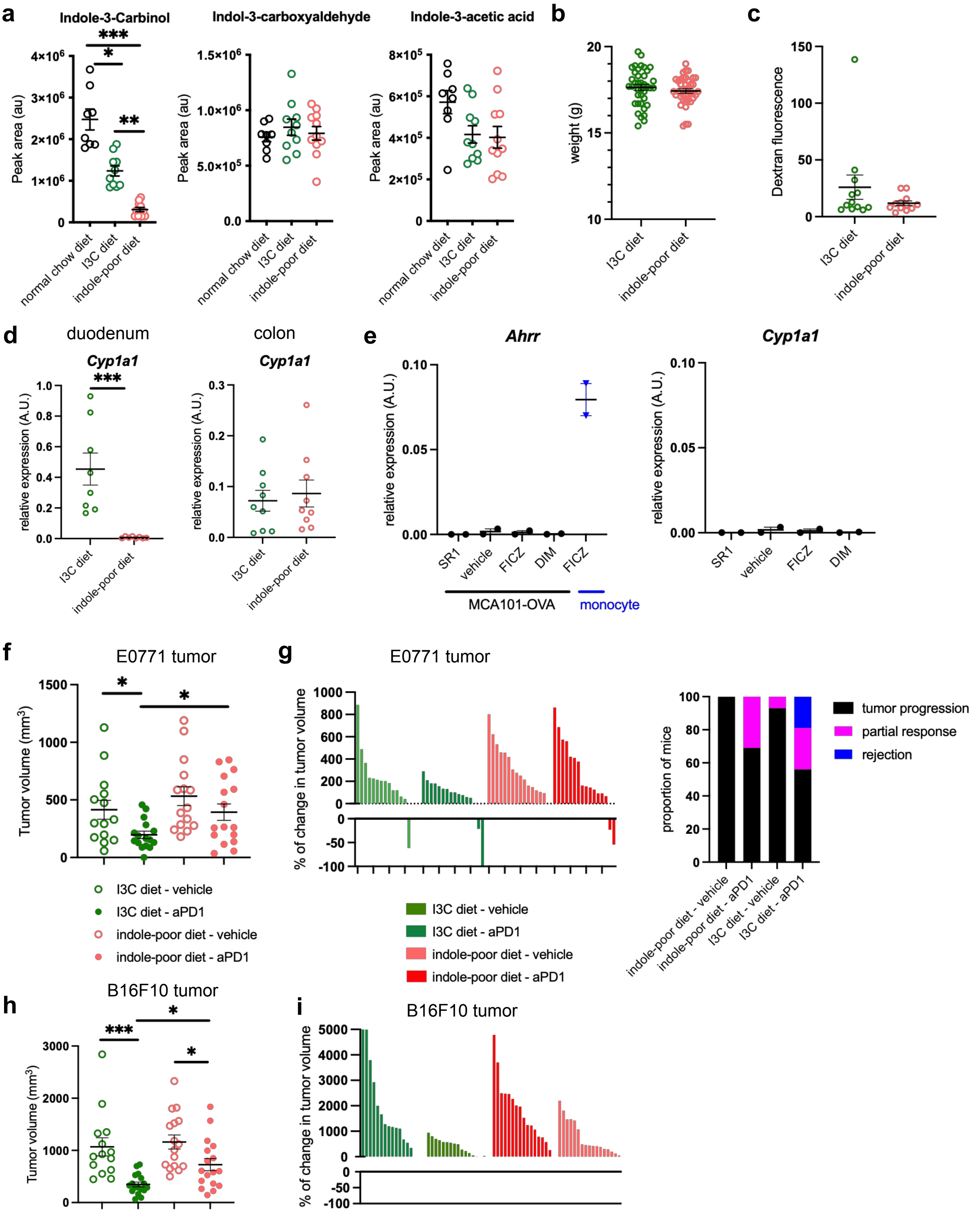

Supplementary Fig. 1. Dietary AhR ligands are essential for the efficacy of anti-PD1 immunotherapy in preclinical models. Mice were placed on standard chow diet (a), indole-poor diet or I3C diet (a-d, f-h) for 3 weeks of adaptation prior to the start of experiments. (a) Relative amount of indicated metabolites was measured in serum. Mean +/- sem are shown (n = 8–11 in two independent experiments). One-way ANOVA. (b) Mouse weight on the day of tumor inoculation. Mean +/- sem are shown

(n=39 biological replicates in 6 independent experiments). Mann-Whitney test. (c) Intestinal permeability was assessed by gavage with fluorescent Dextran. Fluorescence in the plasma was assessed after 2h. Mean +/- sem are shown (n=12 biological replicates in 3 independent experiments). Mann-Whitney test. (d) Intestinal lysates were analyzed by RT-qPCR in naive mice. Mean +/- sem are shown (n=8-9 biological replicates in 2 independent experiments). AU, arbitrary units. Mann-Whitney test. (e) MCA101-OVA cells were exposed in vitro to AhR inhibitor (SR1) or agonists (FICZ or DIM). Relative expression of indicated genes (n=2 independent experiments). Bone marrow monocytes are included as positive control. AU, arbitrary units. (f-g) Mice were inoculated with E0771 tumor cells. When tumors reached 80-100 mm^3, mice were treated intra-peritoneally with 3 doses of anti-PD1 or vehicle. (f) Tumor volume at day 10 after the first treatment dose (n=15-16 in 3 cohorts). One-way ANOVA. (g) Percentage of change in volume for each tumor between day 0 and day 10 of treatment (n=15-16 in 3 cohorts). Proportion of mice showing tumor rejection, partial response, or tumor progression (n=15-16 in 3 cohorts) (h-i) Mice were inoculated with B16F10 tumor cells. When tumors reached 80-100 mm^3, mice were treated intra-peritoneally with 3 doses of anti-PD1 or vehicle. (h) Tumor volume at day 10 after the first treatment dose (n=14-17 in 3 cohorts). One-way ANOVA. (i) Percentage of change in volume for each tumor between day 0 and day 10 of treatment (n=14-17 in 3 cohorts). For all panels, * p<0.05, ** p<0.01, *** p<0.001. Absence of star indicates 'not significant'. Source data are provided as a Source Data file.

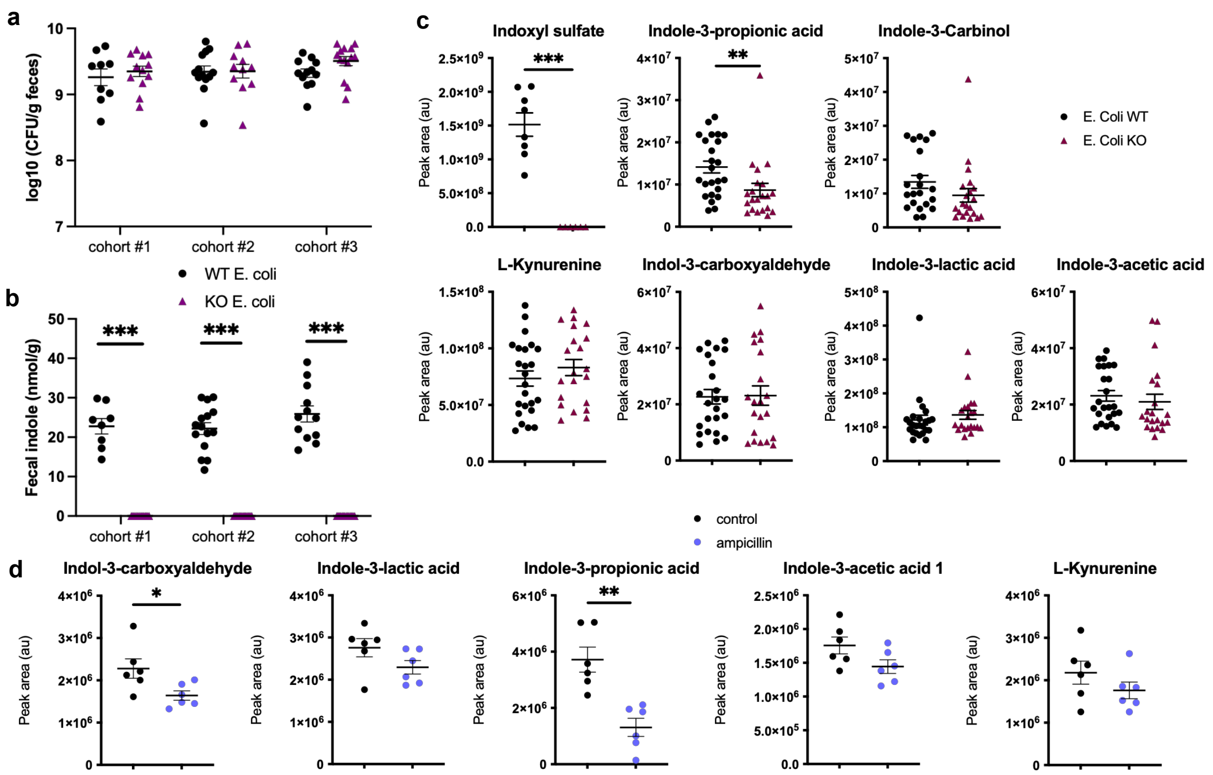

Supplementary Fig. 2. Relative abundance of circulating AhR ligands after microbiota manipulation. (a-c) Germ-free mice were reconstituted with Tryptophanase-competent (WT) or -deficient (KO) *E. coli*. (a) Total bacteria were quantified in feces as a measure for implantation (n=9-14 mice per group, in 3 separate cohorts). (b) Indole was measured in feces (n=9-14 mice per group, in 3 separate cohorts). Two-way ANOVA. (c) Relative amount of metabolites was measured in serum (n=8-24 mice in 2-4 cohorts). Mann-Whitney test. (d) Mice were treated orally with Ampicillin or vehicle control. Relative amount of metabolites was measured in serum. Mean +/- sem are shown (n=6 mice in 1 cohort). Mann-Whitney test. For all panels, * $p<0.05$, ** $p<0.01$, *** $p<0.001$. Absence of star indicates 'not significant'. Source data are provided as a Source Data file.

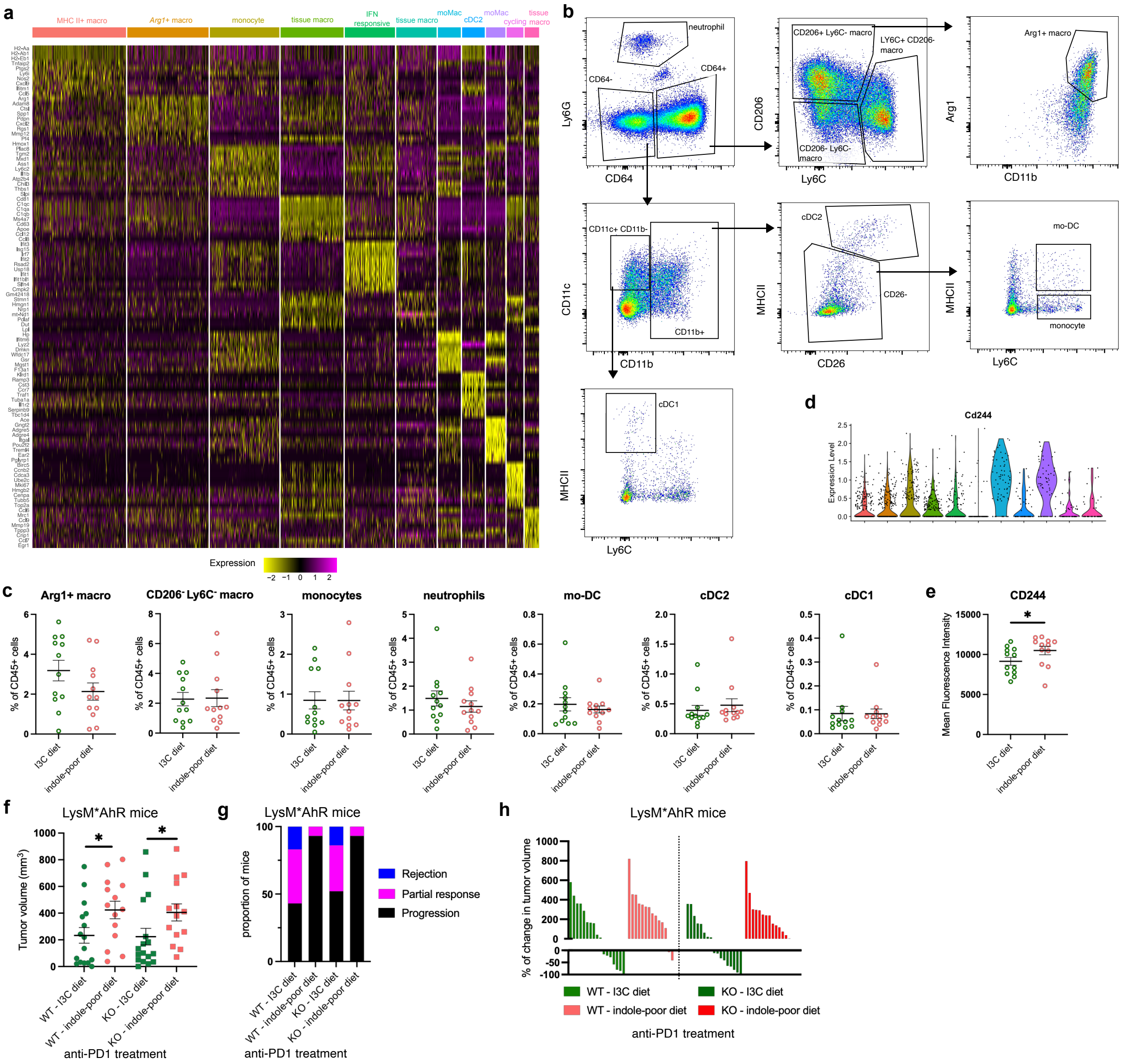

Supplementary Fig. 3. AhR activation in macrophages does not affect responses to anti-PD1 therapy. (a-e) Mice were placed on indole-poor diet or enriched in I3C diet for 3 weeks of adaptation prior to the start of experiments. Mice were inoculated with MCA101-OVA tumor cells. Tumors were analyzed when they reached 80-100 mm^3. (a and d) Tumor-infiltrating CD11b+ cells were analyzed by scRNA-seq (n=2 biological replicates per group). (a) Top differentially expressed genes across clusters. (b-c and e) Cells were analyzed by flow cytometry. (b) Gating strategy is shown. Gated on single live CD45+ cells. (c) Proportion of indicated populations among live CD45+ cells. Mean +/- sem are shown (n=12 biological replicates in 2 independent experiments). Mann-Whitney test. (d) Expression of *Cd244* gene across scRNA-seq clusters. Clusters are identified with the same color code as in panel A. (e) Mean fluorescence intensity of CD244 on moMac. Mean +/- sem are shown (n=12 biological replicates in 2 independent experiments). Mann-Whitney test. (f-h) LysM*AhR$^{\Delta}$ mice (KO) and WT littermates were used. When tumors reached 80-100 mm^3, mice were treated intra-peritoneally with 3 doses of anti-PD1. (f) Tumor volume at day 10 after the first treatment dose. Mean +/- sem are shown (n=14-17 in 3 cohorts). Kruskall-Wallis test. (g) Proportion of mice showing tumor rejection, partial response, or tumor

progression (n=14-17 in 3 cohorts). (h) Percentage of change in volume for each tumor between day 0 and day 10 of treatment (n=14-17 in 3 cohorts).
For all panels, * $p<0.05$. Absence of star indicates 'not significant'. Source data are provided as a Source Data file.

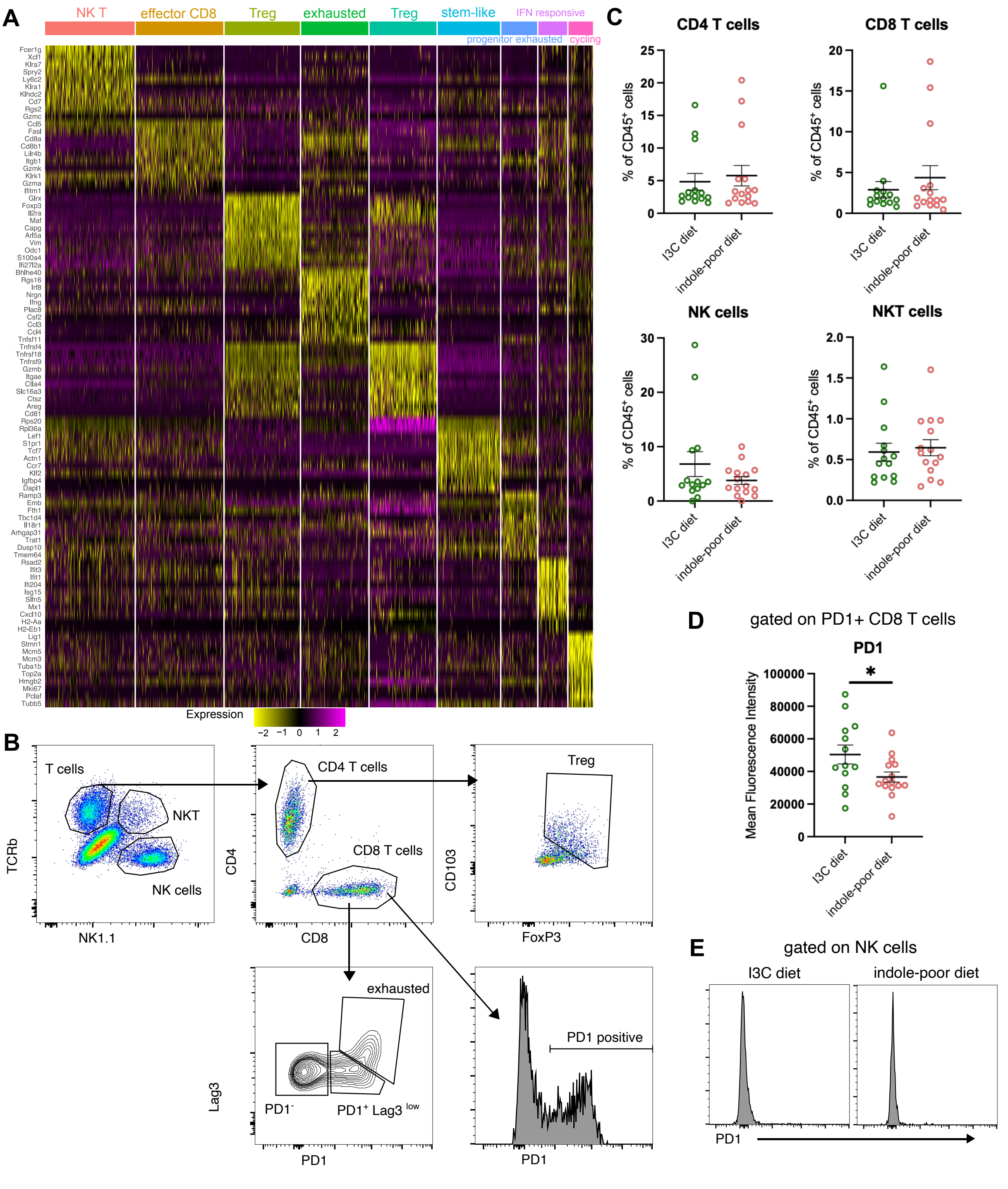

Supplementary Fig. 4. Lack of dietary AhR ligands does not significantly affect the baseline tumor T cells landscape. Mice were placed on indole-poor diet or I3C diet for 3 weeks of adaptation prior to the start of experiments. Mice were inoculated with MCA101-OVA tumor cells. Tumors were analyzed when they reached 80-100 mm^3. (a) Tumor-infiltrating TCRb+ cells were analyzed by scRNA-seq (n=2 biological replicates per group). Top differentially expressed genes across clusters. (b-e) Cells were analyzed by flow cytometry. (b) Gating strategy is shown. Gated on single live CD45+ cells. (c) Proportion of indicated populations among live CD45+ cells. Mean +/- sem are shown (n=12 biological replicates in 2 independent experiments). Mann-Whitney test. (d) Mean fluorescence intensity of anti-PD1 staining in PD1-expressing CD8 T cells. Mean +/- sem are shown (n=13-14 biological replicates in 3 independent

experiments). Mann-Whitney test. (e) Representative stainings for PD1 on NK cells. (n=12 biological replicates in 2 independent experiments).
For all panels, * $p<0.05$. Absence of star indicates 'not significant'. Source data are provided as a Source Data file.

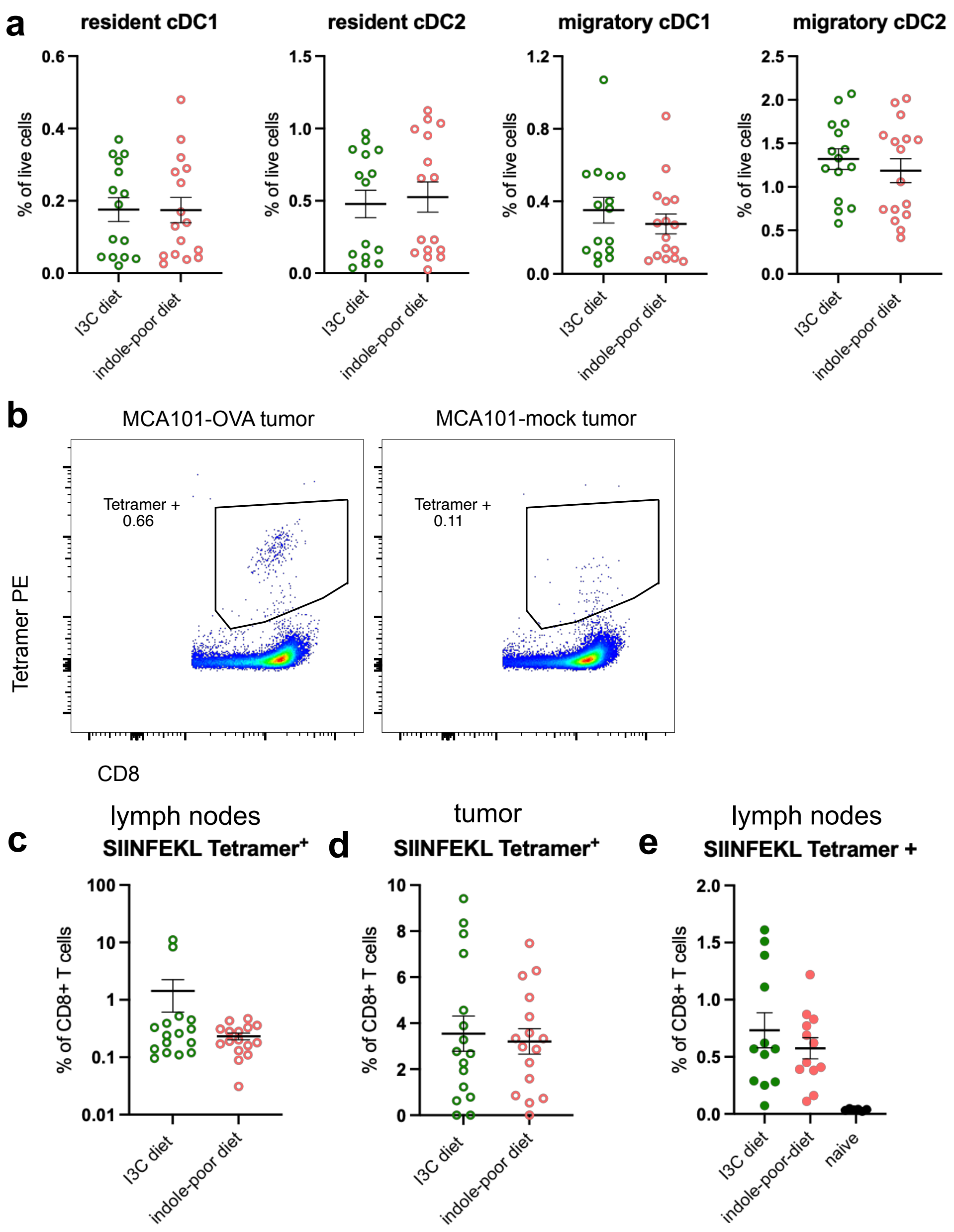

Supplementary Fig. 5. **Dietary AhR ligands do not impact tumor antigen-specific immune responses.** Mice were placed on indole-poor diet or in I3C diet for 3 weeks of adaptation prior to the start of experiments. Mice were inoculated with MCA101-OVA tumor cells. (a-d) When tumors reached 80-100 mm^3, tumor-draining lymph nodes or tumors were analyzed. (a) Percentage of indicated cell populations in lymph nodes among live cells. Mean +/- sem are shown (n=15-16 biological replicates in 3 cohorts). Mann-Whitney test. Dendritic cells were gated as $TCRb^-CD11c^+MHCII^+$. cDC1 and cDC2 were separated based on XCR1 and CD11b. Migratory and resident cells were gated based on MHCII and CD11c expression level. (b-d) Cells were stained for SIINFEKL tetramer. Cells were gated as $TCR\beta^+CD8^+$. (b) Representative tetramer staining on CD8 T cells. Lymph nodes from mice inoculated with MCA101-mock cells were used for comparison. (c) Proportion of tetramer$^+$ CD8 T cells in lymph nodes. Mean +/- sem are shown (n=16 biological replicates in 3 cohorts). Mann-Whitney test. (d) Proportion of tetramer$^+$ CD8 T cells in tumors. Mean +/- sem are shown (n=16 biological replicates). Mann-Whitney test. (e) When tumors reached 80-

100 mm^3, mice were treated with anti-PD1. Proportion of tetramer$^+$ CD8 T cells in tumor-draining lymph nodes 48h after treatment. Mean +/- sem are shown (n=12 biological replicates in 3 cohorts). Lymph nodes from naive mice (no tumor) were included for assessing background levels. Kruskall-Wallis test.
Absence of star indicates 'not significant'. Source data are provided as a Source Data file.

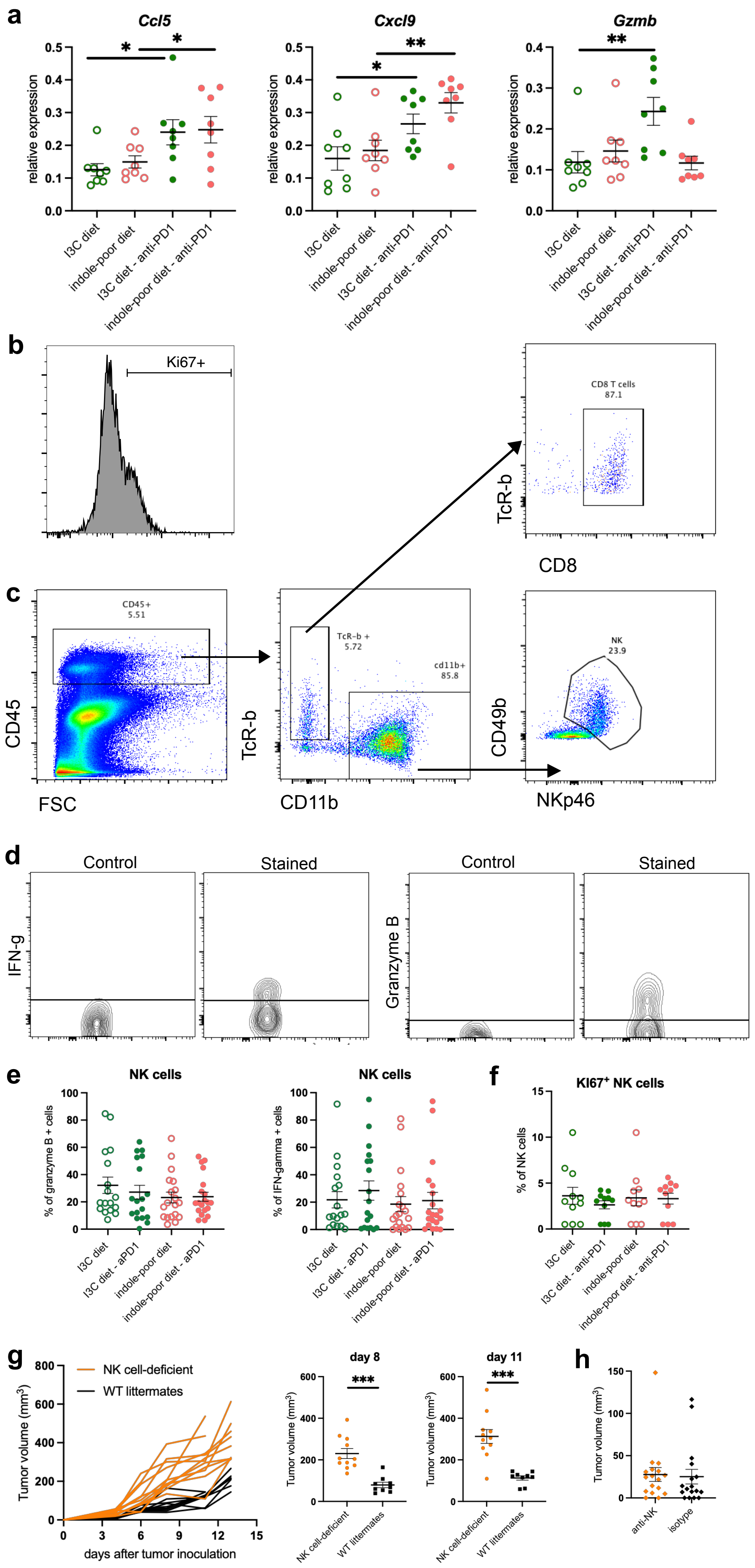
a
Ccl5
Cxcl9
Gzmb
relative expression
I3C diet
indole-poor diet
I3C diet - anti-PD1
indole-poor diet - anti-PD1
b
Ki67+
CD8 T cells
87.1
TcR-b
CD8
c
CD45+
5.51
CD45
FSC
TcR-b +
5.72
cd11b+
85.8
TcR-b
CD11b
NK
23.9
CD49b
NKp46
d
Control
Stained
IFN-g
Granzyme B
e
NK cells
% of granzyme B + cells
% of IFN-gamma + cells
I3C diet - aPD1
indole-poor diet - aPD1
f
KI67+ NK cells
% of NK cells
g
NK cell-deficient
WT littermates
Tumor volume (mm3)
days after tumor inoculation
day 8
day 11
h
anti-NK
isotype

Supplementary Fig. 6. Lack of dietary AhR ligands impairs NK cells infiltration without affecting granzyme B and IFN-γ production. Mice were inoculated with MCA101-OVA tumor cells. Mice were treated when tumors reached 80-100 mm^3. (a-f) Mice were placed on indole-poor diet or I3C diet for 3 weeks of adaptation prior to the start of experiments. (a) Tumors were analyzed either on the day of injection (no treatment) or 24h after anti-PD1 injection. Tumor-infiltrating immune cells were analyzed by RT-qPCR. Relative expression of indicated genes (n=8 biological replicates). One-way ANOVA. (b-f) Tumor-infiltrating immune cells were analyzed by flow cytometry. (b) Gating strategy for analyzing Ki67 staining, after gating CD8 T cells as in Supplementary Fig. 3C. (c) Gating strategy for quantifying tumor-infiltrating CD8 T cells and NK cells and analyzing intracellular staining. Cells were gated on live single cells. (d) Gating strategy for analyzing IFN-γ and granzyme B expression by intracellular staining. Control condition corresponds to the absence of antibodies for intracellular staining. (e) Percentage of granzyme B+ or IFN-γ+ cells among NK cells. (f) Percentage of Ki67+ cells among NK cells. (g) Ncr1*Il2rg$^{\Delta}$ mice and WT littermates, fed on normal chow diet, were used. Tumor growth kinetics. Tumor volume at indicated time points. Mean +/- sem are shown (n=9-11 in 2 cohorts). Unpaired t-test. (h) C57Bl/6 mice were used. Mice were treated intra-peritoneally with 6 doses of anti-NK1.1 or control antibody, and 3 doses of anti-PD1 or vehicle. Tumor volume at day 10 after the first treatment dose Mean +/- sem are shown (n=16-17 in 3 cohorts). Mann-Whitney test.

For all panels, * $p<0.05$, ** $p<0.01$, *** $p<0.001$. Absence of star indicates 'not significant'. Source data are provided as a Source Data file.

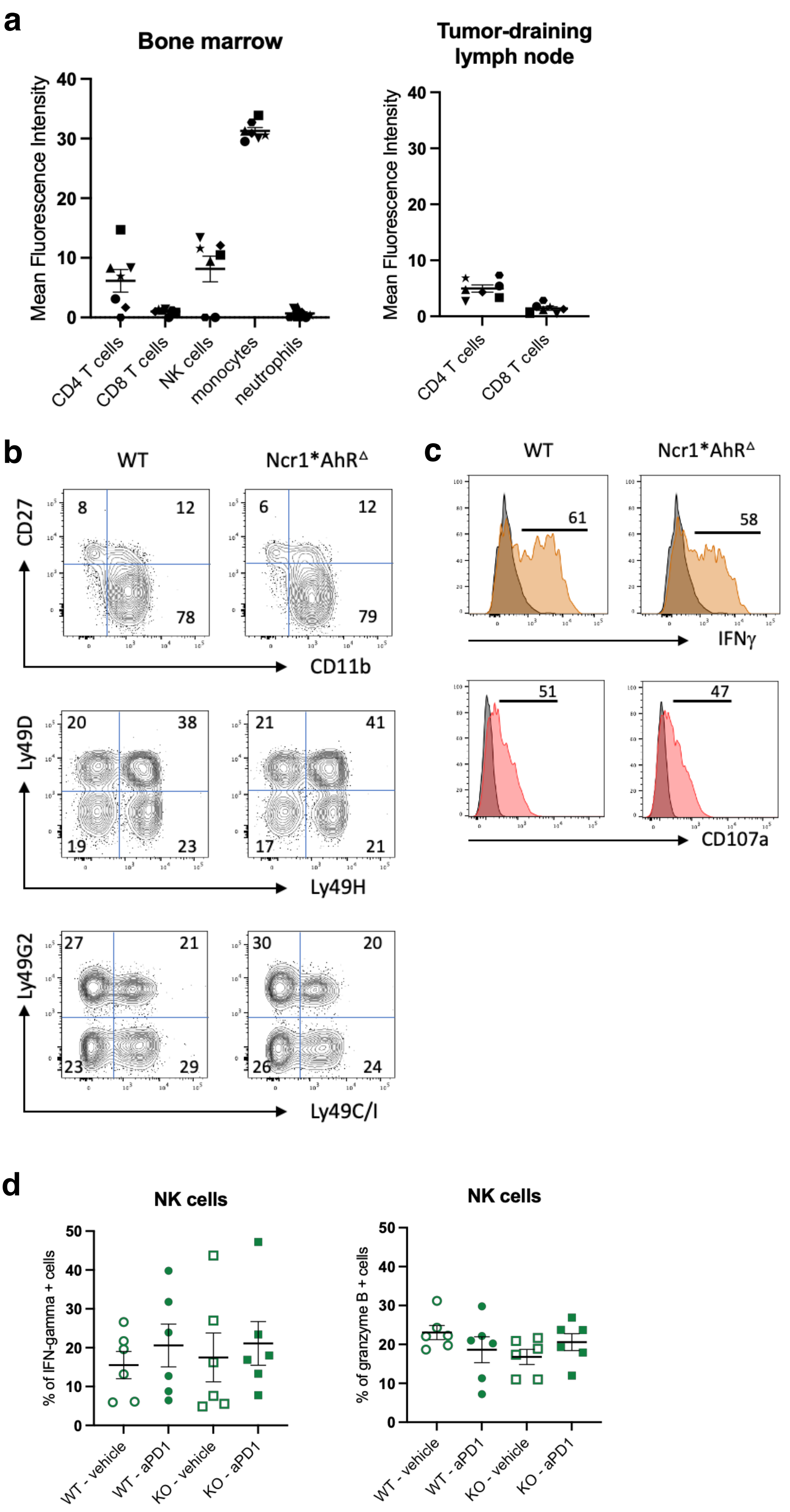

Supplementary Fig. 7. AhR deficiency in NK cells does not impact their functional properties. (a) AhR-TdTomato reporter mice were used. Mice were

analyzed when tumors reached 80-100 mm^3. Mean Fluorescence Intensity of TdTomato of indicated cell populations in bone marrow or tumor-draining lymph nodes (n= 7 biological replicates in 2 cohorts). Each symbol represents one individual mouse. (b-d) Ncr1*AhR$^{\Delta}$ mice (KO) and WT littermates were used. (b) Spleen NK cells were analyzed by flow cytometry for indicated phenotypic markers. Gated on live single $CD3^{-}CD5^{-}CD19^{-}$ cells. Representative results are shown (n=3 independent experiments). (c) Spleen NK cells were stimulated *ex vivo* with IL-12 and IL-18 (upper panel) or PMA and ionomycin (lower panel) for 4hrs in the presence of brefeldin A. Intracellular expression of IFNγ (upper panel) and surface expression of CD107a (lower panel) is shown. Gray shaded histograms represent unstimulated cells. Gated on live single $CD3^{-}CD5^{-}CD19^{-}$ cells. Numbers indicate frequencies of the gated populations. Representative results are shown (n=3 independent experiments). (d) Mice were placed on I3C diet for 3 weeks of adaptation prior to the start of experiments. Mice were inoculated with MCA101-OVA tumor cells and treated when tumors reached 80-100 mm^3. Tumor-infiltrating immune cells were analyzed by intracellular flow cytometry, 48h after vehicle or anti-PD1 injection. Percentage of granzyme B+ or IFN-γ+ cells among NK cells. Mean +/- sem are shown (n=6 biological replicates in 2 independent experiments). One-way ANOVA. Absence of star indicates 'not significant'. Source data are provided as a Source Data file.

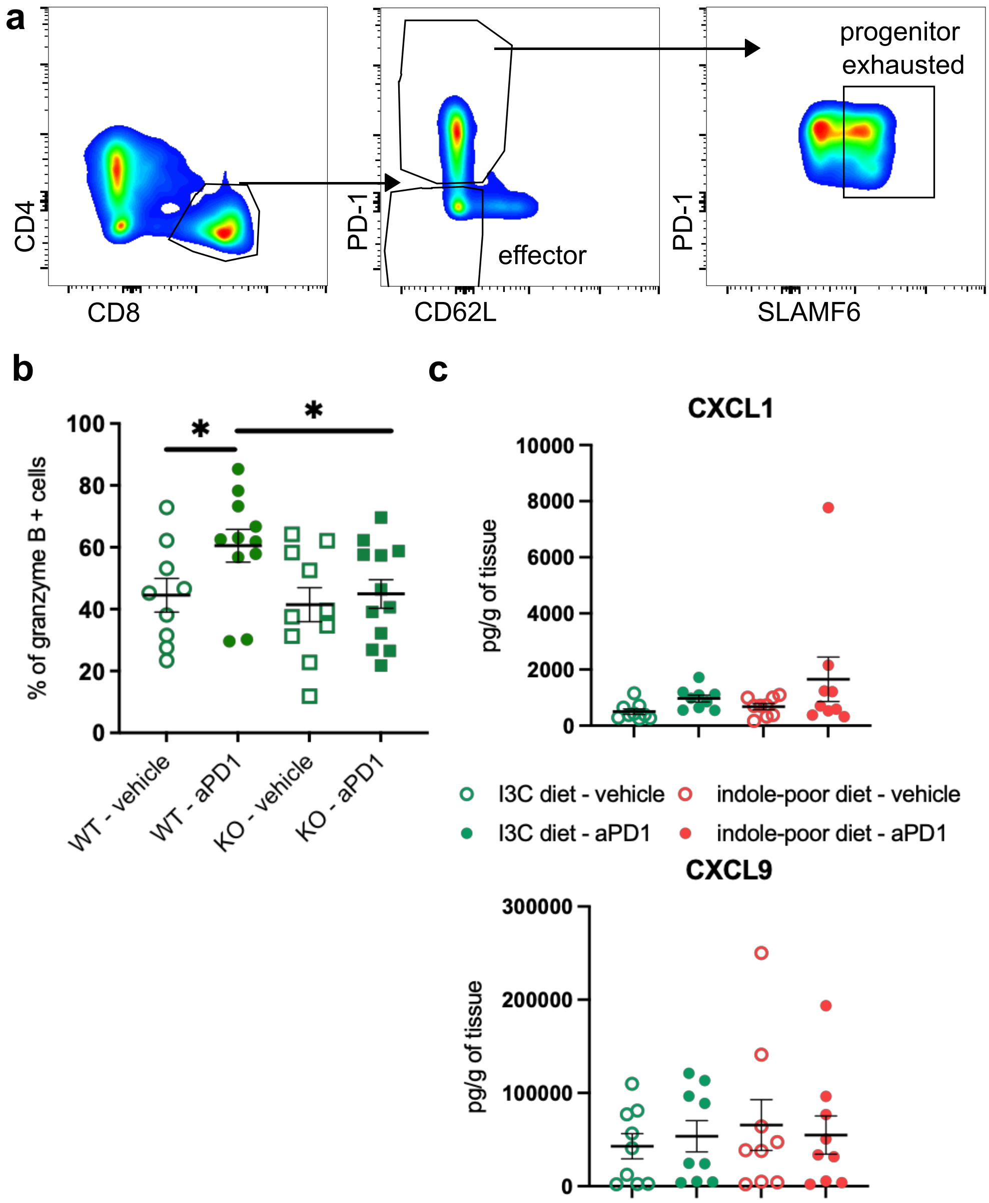

Supplementary Fig. 8. AhR regulates part of the functional response of effector CD8 T cells to anti-PD1 treatment. Mice were inoculated with MCA101-OVA tumor cells. Mice were treated when tumors reached 80-100 mm^3. (a) Gating strategy for analysis of tumor-infiltrating ‘progenitor exhausted’ and ‘effector’ CD8 T cells. Gated on live CD11b- TCR-β+ cells. (b) Lck*AhR^{Δ} mice (KO) and WT littermates were placed on I3C diet for 3 weeks of adaptation prior to the start of experiments. Tumor-infiltrating immune cells were analyzed by flow cytometry for granzyme B expression, 48h after one dose of vehicle or anti-PD1. Mean +/- sem are shown (n=9-12 biological replicates in 3 cohorts). Kruskall-Wallis test. (c) Mice were placed on indole-poor diet or enriched in Indole-3-carbinol (I3C diet) for 3 weeks of adaptation. Tumors were lysed 48h after anti-PD1 injection. Chemokine concentration was assessed by cytometric bead array. Mean +/- sem are shown (n=9 biological replicates in 3 cohorts).

For all panels, * $p<0.05$. Absence of star indicates 'not significant'. Source data are provided as a Source Data file.
