# Supplementary material for: Physiological activation of Aryl hydrocarbon receptor by food-derived ligands is essential for the efficacy of anti-PD1 therapy
Source: Nat Commun. 2025 Dec 2;16:10598. doi: 10.1038/s41467-025-66854-x (PMC12672584; doi:10.1038/s41467-025-66854-x)
Supplement: Supplementary file 2 — Description of Additional Supplementary Files [file 41467_2025_66854_MOESM2_ESM.pdf]

Supplementary data

Supplementary data 1. List of differentially expressed genes between WT and AhR-deficient cells for 'progenitor exhausted' CD8 T cells (upregulated in WT). lfcSE: standard error estimate for the log2 fold change. p value from one-sided Wald test. padj: adjusted p value for multiple testing using the procedure of Benjamini and Hochberg.

Supplementary data 2. List of differentially expressed genes between WT and AhR-deficient cells for 'progenitor exhausted' CD8 T cells (upregulated in KO). lfcSE: standard error estimate for the log2 fold change. p value from one-sided Wald test. padj: adjusted p value for multiple testing using the procedure of Benjamini and Hochberg.

Supplementary data 3. List of differentially expressed genes between WT and AhR-deficient cells for effector CD8 T cells (upregulated in WT). lfcSE: standard error estimate for the log2 fold change. p value from one-sided Wald test. padj: adjusted p value for multiple testing using the procedure of Benjamini and Hochberg.

Supplementary data 4. List of differentially expressed genes between WT and AhR-deficient cells for effector CD8 T cells (upregulated in KO). lfcSE: standard error estimate for the log2 fold change. p value from one-sided Wald test. padj: adjusted p value for multiple testing using the procedure of Benjamini and Hochberg.

Supplementary data 5. List of antibodies used for flow cytometry panels.
