# Supplementary material for: Physiological activation of Aryl hydrocarbon receptor by food-derived ligands is essential for the efficacy of anti-PD1 therapy
Source: Nat Commun. 2025 Dec 2;16:10598. doi: 10.1038/s41467-025-66854-x (PMC12672584; doi:10.1038/s41467-025-66854-x)
Supplement: Supplementary file 8 — Reporting Summary [file 41467_2025_66854_MOESM8_ESM.pdf]

nature portfolio

Corresponding author(s): Elodie SEGURA

Last updated by author(s): Oct 28, 2025

Reporting Summary

Nature Portfolio wishes to improve the reproducibility of the work that we publish. This form provides structure for consistency and transparency in reporting. For further information on Nature Portfolio policies, see our Editorial Policies and the Editorial Policy Checklist.

Statistics

For all statistical analyses, confirm that the following items are present in the figure legend, table legend, main text, or Methods section.

n/a	Confirmed	
[ ]	[x]	The exact sample size (*n*) for each experimental group/condition, given as a discrete number and unit of measurement
[ ]	[x]	A statement on whether measurements were taken from distinct samples or whether the same sample was measured repeatedly
[ ]	[x]	The statistical test(s) used AND whether they are one- or two-sided<br>*Only common tests should be described solely by name; describe more complex techniques in the Methods section.*
[x]	[ ]	A description of all covariates tested
[ ]	[x]	A description of any assumptions or corrections, such as tests of normality and adjustment for multiple comparisons
[x]	[ ]	A full description of the statistical parameters including central tendency (e.g. means) or other basic estimates (e.g. regression coefficient) AND variation (e.g. standard deviation) or associated estimates of uncertainty (e.g. confidence intervals)
[ ]	[x]	For null hypothesis testing, the test statistic (e.g. *F*, *t*, *r*) with confidence intervals, effect sizes, degrees of freedom and *P* value noted<br>*Give P values as exact values whenever suitable.*
[x]	[ ]	For Bayesian analysis, information on the choice of priors and Markov chain Monte Carlo settings
[x]	[ ]	For hierarchical and complex designs, identification of the appropriate level for tests and full reporting of outcomes
[x]	[ ]	Estimates of effect sizes (e.g. Cohen's *d*, Pearson's *r*), indicating how they were calculated

Our web collection on statistics for biologists contains articles on many of the points above.

Software and code

Policy information about availability of computer code

Data collection	n/a
Data analysis	Statistical tests were performed using Prism v10 (GraphPad Software).

For manuscripts utilizing custom algorithms or software that are central to the research but not yet described in published literature, software must be made available to editors and reviewers. We strongly encourage code deposition in a community repository (e.g. GitHub). See the Nature Portfolio guidelines for submitting code & software for further information.

Data

Policy information about availability of data

All manuscripts must include a data availability statement. This statement should provide the following information, where applicable:

- Accession codes, unique identifiers, or web links for publicly available datasets
- A description of any restrictions on data availability
- For clinical datasets or third party data, please ensure that the statement adheres to our policy

Sequencing data generated during the study is available at GEO (GSE271049 for RNA-seq and GSE271049 for scRNA-seq). Other data that support the findings of this study are available from the corresponding author upon reasonable request.

Research involving human participants, their data, or biological material

Policy information about studies with human participants or human data. See also policy information about sex, gender (identity/presentation), and sexual orientation and race, ethnicity and racism.

Reporting on sex and gender	n/a
Reporting on race, ethnicity, or other socially relevant groupings	n/a
Population characteristics	n/a
Recruitment	n/a
Ethics oversight	n/a

Note that full information on the approval of the study protocol must also be provided in the manuscript.

Field-specific reporting

Please select the one below that is the best fit for your research. If you are not sure, read the appropriate sections before making your selection.

[x] Life sciences   [ ] Behavioural & social sciences   [ ] Ecological, evolutionary & environmental sciences

For a reference copy of the document with all sections, see nature.com/documents/nr-reporting-summary-flat.pdf

Life sciences study design

All studies must disclose on these points even when the disclosure is negative.

Sample size	Sample-size calculation was performed using InVivo stat software (v4.2). Experiments were replicated independently.
Data exclusions	No data was excluded.
Replication	The number of biological replicates and of independent experiments is indicated in each legend. For all experiments in this manuscript, samples or animals were allocated to experiments and experimental groups randomly.
Randomization	No randomization method was used.
Blinding	For all experiments, investigators were blinded during data collection by giving each sample analyzed an arbitrary number. Blinding was not possible for analyzing tumor growth kinetics, because measurements were performed longitudinally for each mouse.

Reporting for specific materials, systems and methods

We require information from authors about some types of materials, experimental systems and methods used in many studies. Here, indicate whether each material, system or method listed is relevant to your study. If you are not sure if a list item applies to your research, read the appropriate section before selecting a response.

Materials & experimental systems

n/a	Involved in the study	
[ ]	[x]	Antibodies
[ ]	[x]	Eukaryotic cell lines
[x]	[ ]	Palaeontology and archaeology
[ ]	[x]	Animals and other organisms
[x]	[ ]	Clinical data
[x]	[ ]	Dual use research of concern
[x]	[ ]	Plants

Methods

n/a	Involved in the study	
[x]	[ ]	ChIP-seq
[ ]	[x]	Flow cytometry
[x]	[ ]	MRI-based neuroimaging

Antibodies

Antibodies used	For in vivo immunotherapy, mice were treated with anti-PD1 (BioXcell).<br>For in vivo NK cell depletion, mice were treated with anti-mouse NK1.1 (clone PK136) or of isotype control antibody (both from BioXcell).<br>Flow cytometry of tumors. Myeloid panel: anti-CD26 BV605 (Biolegend, clone DPP-4), anti-Ly6G BV510 (Biolegend, clone 1A8), anti-CD11c BV786 (BD Biosciences, clone HL3), anti-Ly6C Alexa700 (Biolegend, clone HK1.4), anti-MHC II Pe-Cy5 (Biolegend, clone

M5/114.15.2), anti-CD64 PerCP-eFluor710 (eBioscience, clone X54-5/7.1), anti-CD206 BV650 (Biolegend, clone C068C2), anti-Arg1 APC (eBioscience, clone A1exF5), anti-CD11b BV750 (BD Bioscience, clone M1/70), anti-CD45 AF532 (eBioscience, clone 30-F11). Lymphoid panel: anti-NK1.1 BV605 (BD Bioscience, clone PK136), anti-TCR FITC (BD Bioscience, clone H57-597), anti-CD8 BV711 (BD Bioscience, clone 53-6.7), anti-CD4 BV650 (Biolegend, clone GK1.5), anti-CD103 PerCP-Cy5.5 (Biolegend, clone 2E7), anti-Foxp3 APC (eBioscience, clone FJK-16s), anti-Lag3 PE-Dazzle594 (Biolegend, clone C9B7W), anti-PD1 BV421 (Biolegend, clone 29F.1A12), anti-CD45 AF532 (eBioscience, clone 30-F11). Samples were acquired on a Aurora instrument (Cytek).

For tetramer staining, cells were stained with H-2 Kb SIINFEKL Tetramer PE (MBL international) and anti-CD45 V500 (BD Bioscience, clone 30-F11), anti-CD8 APC-Cy7 (BD Pharmingen, clone 53-6-7), anti-TCR FITC (BD Bioscience, clone H57-597), anti-CD4 PerCP-Cy5.5 (Biolegend, clone GK1.5). Samples were acquired on a FACSVerse instrument (BD Bioscience).

For cytokine intracellular staining, cells were stained with anti-CD8 BUV 395 (BD Bioscience, clone 53-6.7), anti-TCR BUV737 (BD Bioscience, clone H57-597), anti-CD45 FITC (BD Biosciences, clone HI30), anti-NKp46 BV605 (Biolegend, clone 29A1.4), anti-CD11b PE-CF594 (BD Bioscience, clone M1/70), anti-CD49b APC (BD Bioscience, clone DX5), anti-Granzyme B BV421 (BD Bioscience, clone GB11), anti-IFN BV785 (Biolegend, clone XMG1.2). Samples were acquired on a ZE5 instrument (Biorad).

For the analysis of AhR-TdTomato mice, cells were stained with anti-TCR BUV737 (BD Bioscience, clone H57-597), anti-CD8 BUV 395 (BD Bioscience, clone 53-6.7), anti-NK1.1 BV480 (BD Bioscience, clone PK136), anti-CD11b PerCP-Cy5.5 (BD Bioscience, clone M1/70), anti-Ly6G BV605 (Biolegend, clone 1A8), anti-CD11c BV786 (BD Biosciences, clone HL3), anti-CD45 FITC (BD Biosciences, clone HI30), anti-CD26 PE (Biolegend, clone DPP-4), anti-Ly6C Alexa700 (Biolegend, clone HK1.4), anti-MHC II APC-Cy7 (Biolegend, clone M5/114.15.2), anti-CD64 Pe-Cy7 (eBioscience, clone X54-5/7.1), anti-CD49b APC (BD Bioscience, clone DX5). Samples were acquired on a ZE5 instrument (Biorad).

For the analysis of 'progenitor exhausted' and 'effector' CD8+ T cells, cells were stained with anti-TCR BUV395 (BD Bioscience, clone H57-597), anti-CD11b BV750 (Biolegend, clone M1/70), anti-PD1 BV421 (Biolegend, clone 29F.1A12), anti-CD278/ICOS BUV737 (BD Bioscience, clone C398.4A), anti-CD25 BV605 (Biolegend, clone PC61), anti-CD45 Alexa Fluor 532 (eBioscience, clone HI30), anti-CD4 PerCP-Cy5.5 (BD Bioscience, clone RM4-5), anti-CD62L APC (Biolegend, clone MEL-14), anti-CD357/GITR Pe-Cy7 (eBioscience, clone eBioAITR), anti-Slamf6 PE (Biolegend, clone 330-AJ), anti-CD8 APC-Cy7 (Biolegend, clone YTS156.7.7). Samples were acquired on an Aurora instrument (Cytek).

Flow cytometry of lymph nodes. For analysis prior to therapy, cells were stained with anti-TCR PeCy7 (BD Bioscience, clone H57-597), anti-CD8 APC (BD Pharmingen, clone 53-6-7), anti-CD11c FITC (BD Pharmingen, clone H13), anti-XCR1 PerCP-Cy5.5 (Biolegend, clone ZET), anti-CD103 BV510 (Biolegend, clone 2E7), anti-CD11b PE-Texas Red (BD Bioscience, clone M1/70), anti-MHC II APC Cy7 (Biolegend, clone M5/114.15.2). Samples were acquired on a FACSVerse instrument (BD Bioscience). For analysis after therapy, cells were stained with anti-TCR BUV737 (BD Bioscience, clone H57-597), anti-CD8 BUV 395 (BD Bioscience, clone 53-6.7), anti-CD19 BV480 (BD Bioscience, clone 1D3), anti-CD11b PerCP-Cy5.5 (BD Bioscience, clone M1/70). Samples were acquired on a ZE5 instrument (Biorad). For the analysis of AhR-TdTomato mice, cells were stained with anti-TCR BUV737 (BD Bioscience, clone H57-597), anti-CD8 BUV395 (BD Bioscience, clone 53-6.7), anti-CD19 BV480 (BD Bioscience, clone 1D3), anti-CD4 PerCP-Cy5.5 (BD Bioscience, clone RM4-5), anti-CD11b Pe-Cy7 (BD Bioscience, clone M1/70). Samples were acquired on a ZE5 instrument (Biorad).

Flow cytometry of bone marrow cells. cells were stained with anti-TCR BUV737 (BD Bioscience, clone H57-597), anti-CD8 BUV395 (BD Bioscience, clone 53-6.7), anti-NK1.1 BV480 (BD Bioscience, clone PK136), anti-CD11b Pe-Cy7 (BD Bioscience, clone M1/70), anti-Ly6G BV605 (Biolegend, clone 1A8), anti-Ly6C FITC (Biolegend, clone HK1.4), anti-CD115 APC (BD Bioscience, clone AFS98), anti-CD43 BB700 (BD Bioscience, clone S7). Samples were acquired on a ZE5 instrument (Biorad).

Flow cytometry of spleen NK cells. Cells were stained with anti-CD3 BV605 (BD Biosciences, clone 145-2C11), anti-CD5 APC-R700 (BD Biosciences, clone 53-7.3), anti-CD11b FITC (eBioscience, clone M1/70), anti-CD19 BV786 (BD Biosciences, clone 1D3), anti-CD27 V450 (BD Biosciences, clone LG3.A10), anti-CD45 BUV395 (BD Biosciences, clone 30F11), anti-Ly49H Pe-CF594 (BD Biosciences, clone 3D11), anti-Ly49D APC (eBioscience, clone 4E5), anti-NKp46 PerCP-eFluor710 (eBioscience, clone 29A1.4), anti-Ly49G2 Pe-Cy7 (BD Biosciences, clone 4D11), anti-Ly49C/I PE (BD Biosciences, clone 5E6).

Validation	Antibodies were validated by the manufacterers.

Eukaryotic cell lines

Policy information about cell lines and Sex and Gender in Research

Cell line source(s)	MCA101-OVA fibrosarcoma cells; E0771 mammary tumor cells ; B16F10 melanoma cells. All cell lines were obtained in-house
Authentication	None of the cell lines were authenticated
Mycoplasma contamination	All cell lines were negative for mycoplasma contamination
Commonly misidentified lines (See ICLAC register)	*Name any commonly misidentified cell lines used in the study and provide a rationale for their use.*

Animals and other research organisms

Policy information about studies involving animals; ARRIVE guidelines recommended for reporting animal research, and Sex and Gender in Research

Laboratory animals	C57BL/6J mice (strain 000664) were obtained from Charles River (France). Lysm-Cre (strain 004781), Lck-Cre (strain 003802) and Ahr-flox (strain 006203) mice were obtained from Jackson Laboratory. Ncr1-iCre mice and Ncr1*Il2rg mice were maintained in-house at Institut Pasteur. Lysm*AhR, Lck*AhR and Ncr1*AhR strains were generated in-house by crossing Ahr-flox with Lysm-Cre Lck-Cre or Ncr1-iCre mice, respectively. AhR-Tomato reporter mice (strain BSEC AHR Tomato) were obtained from B.Stockinger (King's College London) and have been described previously. All mice were on C57BL/6 background.
Wild animals	n/a

Reporting on sex	Only female mice were used.
Field-collected samples	n/a
Ethics oversight	All animal procedures were in accordance with the guidelines and regulations of the French Veterinary Department and have been approved by the local ethics committee (CEEA118 Comité Recherche et Ethique Animale de l'institut Curie, authorization APAFiS#14331-20180330114641-v2).

Note that full information on the approval of the study protocol must also be provided in the manuscript.

Plants

Seed stocks	n/a
Novel plant genotypes	n/a
Authentication	n/a

Flow Cytometry

Plots

Confirm that:

- [x] The axis labels state the marker and fluorochrome used (e.g. CD4-FITC).
- [x] The axis scales are clearly visible. Include numbers along axes only for bottom left plot of group (a 'group' is an analysis of identical markers).
- [x] All plots are contour plots with outliers or pseudocolor plots.
- [x] A numerical value for number of cells or percentage (with statistics) is provided.

Methodology

Sample preparation	Cells were stained with indicated antibody cocktails (Supplementary Table 1) supplemented with Fc block (#553141, BD Biosciences) in FACS buffer (PBS containing 0.5% BSA and 2mM EDTA) for 30-45 min on ice. After washing with FACS buffer, cells were resuspended in FACS buffer containing DAPI (#D1306, Fischer Scientific, 100ng/mL), unless otherwise indicated.
Instrument	Cells were acquired on a ZE5 instrument (Bio-Rad), Aurora (Cytek) or FACSVerse (BD Bioscience).
Software	Supervised analysis was performed using FlowJo software v10 (FlowJo LLC).
Cell population abundance	n/a
Gating strategy	All gating strategies are shown in supplementary figures.

- [x] Tick this box to confirm that a figure exemplifying the gating strategy is provided in the Supplementary Information.
